# Toxicarioside O Inhibits Cell Proliferation and Epithelial-Mesenchymal Transition by Downregulation of Trop2 in Lung Cancer Cells

**DOI:** 10.3389/fonc.2020.609275

**Published:** 2021-02-04

**Authors:** Wu-Ping Zheng, Feng-Ying Huang, Shu-Zhen Dai, Jin-Yan Wang, Ying-Ying Lin, Yan Sun, Guang-Hong Tan, Yong-Hao Huang

**Affiliations:** The Second Affiliated Hospital of Hainan Medical University, Key Laboratory of Tropical Translational Medicine of Ministry of Education & Hainan Provincial Key Laboratory of Tropical Medicine, Haikou, China

**Keywords:** lung cancer cells, toxicarioside O, cell proliferation, epithelial-mesenchymal transition, trophoblast cell surface antigen 2

## Abstract

Toxicarioside O (TCO), a natural product derived from *Antiaris toxicaria*, has been identified to be a promising anticancer agent. In this study, we aimed to investigate the effect of TCO on the proliferation and epithelial-mesenchymal transition (EMT) of lung cancer cells and its molecular mechanisms. Here, we indicated that TCO inhibits the proliferation of lung cancer cells both *in vitro* and *in vivo*. Our results demonstrated that TCO induces apoptosis in lung cancer cells. Moreover, we found that TCO suppresses EMT program and inhibits cell migration *in vitro*. Mechanistically, TCO decreases the expression of trophoblast cell surface antigen 2 (Trop2), resulting in inhibition of the PI3K/Akt pathway and EMT program. Overexpression of Trop2 rescues TCO-induced inhibition of cell proliferation and EMT. Our findings demonstrate that TCO markedly inhibits cell proliferation and EMT in lung cancer cells and provides guidance for its drug development.

## Introduction

Toxicarioside O (TCO) is one of the cardenolides isolated from the seeds of *Antiaris toxicaria* (1). Traditionally, cardenolidesare has been used for the treatment of congestive heart failure and arrhythmia (2, 3). Recently, accumulating evidence has indicated that cardenolides have significant anticancer effects in various types of human cancer cell (4, 5). Consistent with these findings, TCO exhibits significant cytotoxicity against hepatocellular carcinoma cell line SMMC-7721 and human leukemic cell line K562 (1). We have previously demonstrated that TCO inhibits cell proliferation and induces protective autophagy in colorectal cancer cells (6). However, the effect of TCO on the proliferation and epithelial-mesenchymal transition (EMT) of lung cancer cells and its molecular mechanisms remain largely unknown.

Trophoblast cell surface antigen 2 (Trop2), also known as human tumor-associated calcium signal transducer 2 (Tacstd2), is a surface glycoprotein originally identified in human placental trophoblasts (7, 8), which is seldom expressed in normal tissues but highly expressed in various cancer cells, such as pancreatic cancer (9), gastric cancer (10), lung cancer, and colorectal cancer (11–13). Trop2 overexpression correlates with increased tumor recurrence, invasiveness, and poor clinical outcome, which is considered as a candidate tumor prognostic marker and a promising and important therapeutic target (12, 14–16).

Trop2 is considered to have an important function in the promotion of epithelial-to-mesenchymal transition (EMT). EMT is referred to changes in cell phenotypes from epithelial to mesenchymal states which mediate cancer progression, metastasis, and drug resistance (17–19). It has been reported that Trop2 induces EMT by binding to β-catenin and promotes cancer progression in gastric cancer (20). Trop2 also activates the JAK2/STAT3 pathway to promote EMT formation and metastasis in glioblastoma cells (21). Trop2 overexpression promotes metastasis by inducing EMT in human breast cancer and lung cancer (22, 23). The ability of Trop2 in regulation of EMT indicated that Targeting to Trop2 should be an alternative method to regulate EMT in cancer treatment. In this study, our results indicated that TCO inhibits cell proliferation and EMT in lung cancer cells by downregulation of Trop2, demonstrating the therapeutic potential of TCO for lung cancer treatment.

## Materials and Methods

### Cell Culture, Agents, and Antibodies

Human non-small cell lung carcinoma (NSCLC) cell line A549 and H1299 cells were purchased from the American Type Culture Collection. All cell lines (about in passage number 6–8, mycoplasma-free) were cultured according to the guidelines and maintained in RPMI-1640 supplemented with 10% FBS (Gibco) at 37°C in an atmosphere containing 5% CO_2_. TCO was gifted kindly from Prof. Hao-Fu Dai. The purity of TCO was proved to be ≥95% by chromatographic analysis. TCO was dissolved in dimethyl sulfoxide (100% DMSO) and stored at −20°C for experimental use in this study. A BrdU Cell Proliferation Assay Kit was obtained from Abcam. An AnnexinV-FITC/PI Apoptosis Detection Kit was obtained from KeyGEN Biotech. The following antibodies were used in this study: Trop2 (ab214488) was purchased from Abcam; Cleaved caspase-3 (9661T), Cleaved PARP (5625T), Phospho-Akt (4060S), Akt (8596), Phospho-PI3K p85 (17366), PI3Kp85 (4292), E-cadherin (3195S), N-cadherin (13116S), Vimentin (12826), and Snail (3879) were obtained from Cell Signaling Technology.

### Cell Viability Assay

The cell viability was determined by 3-(4,5-dimethylthiazol-2-yl)-2,5- diphenyltetrazolium bromide (MTT; Sigma, M2128) assay. Briefly, cells (3 × 10^3^ cells/well) were seeded in 96-well plates for 24 h and after treatment, 10 μl of MTT was added to each well and incubated for 4 h. Subsequently, medium was removed and 200 μl of 100% DMSO was added to dissolve the crystal formazan dye. The absorbance value was then determined at 570 nm.

### BrdU Assay

BrdU signaling was determined by a BrdU Cell Proliferation Assay Kit (Abcam, ab126556). Briefly, cells (3 × 10^3^ cells/well) were seeded in 96-well plates and after treatment, BrdU was added and incubated for 12 h. BrdU signaling was then determined by measuring the absorbance at 450 nm.

### Colony Formation Assay

Cells (300 cells/well) were seeded in 24-well plates and treated with the indicated concentration of TCO. After 2 weeks, the colonies were stained with Giemsa for 15 min and washed three times by PBS. The visible colonies were photographed by a Molecular Imager Gel Do XR+ System (Bio-Rad) and counted using Image J software (NIH).

### Flow Cytometry

The indicated cells treated with the tested compounds (0, 1, and 2 μM) for 24 h were harvested and washed with PBS, then resuspended and incubated with PI/Annexin-V solution (KeyGEN Biotech). Apoptosis was analyzed with FACSCalibur flow cytometer (sysmex, CyFlow@ Cube 6, Germany), and data were analyzed by FlowJo7.6.1 software.

### Western Blotting

Cells were harvested and lysed with RIPA buffer (Thermo Fisher Scientific, 89900) and the concentrations of protein were quantified by BCA protein assay kit (Thermo, 23227). Proteins (20 to 60 μg) were separated by SDS-PAGE for 80 min and transferred to PVDF membranes for 90 min (IPVH00010, Merck Millipore, Billerica, MA, USA). Membranes were blocked with a buffer containing Tris (10 mM, pH 7.4), NaCl (150 mM), Tween 20 (0.1%), and bovine serum albumin (5%) for 2 h. After blocking, the membranes were incubated with primary antibodies: Trop2 (1:2,000), Cleaved caspase-3 (1:1,000), Cleaved PARP (1:1,000), Phospho-Akt (1:2,000), Akt (1:1,000), Phospho-PI3K p85 (1:1,000), PI3Kp85 (1:1,000), E-cadherin (1:1,000), N-cadherin (1:1,000), Vimentin (1:1,000), and Snail (1:1,000). Subsequently, the blots were probed with secondary antibodies at room temperature for 2 h. Target proteins were detected by enhanced chemiluminescence reagents (WBKLS0100; Merck Millipore).

### Immunofluorescence

Cells were fixed with 4% paraformaldehyde for 30 min, washed three times with PBS, and then incubated with 0.1% Triton X-100 for permeabilization. Following blocking with 5% BSA, cells were stained with rabbit anti-E-cadherin (1:200) and N-cadherin (1:200) polyclonal antibodies overnight at 4°C and then incubated with Goat Anti-Rabbit IgG H&L (Alexa Fluor® 594) (Abcam, ab150080) for 1 h. Images were captured using a fluorescent microscope (OLYMPUS, BX53).

### Plasmids and Transfection of Cells

CA-Akt (myrAkt delta4-129) was purchased from Addgene (Chengdu, China). The full-length complementary DNA (cDNA) sequences of human Trop2 cDNA were purchased from the Jikai Corporation (Shanghai, China). The full-length cDNA of Trop2 was cloned into pcDNA3.1vector. The plasmids were introduced into cells by Lipofectamine 2000 according to the manufacturer’s instructions. The detailed procedure was performed as described previously (23).

### Wound Healing Assay

Cells (5 × 10^5^ cells/well) were seeded in six-well plates and cultured overnight. The cell monolayers were scratched horizontally using a sterile 100-µl pipette tip. Then, the cells were washed with PBS and cultured in RPMI 1640 medium without FBS. The evaluation of wound healing was done under a light microscope at 24 h after scratching.

### Transwell Migration Assay

Transwell chambers (24-well) (8-µm pore size, Jet Bio-Filtration Co., Ltd, Guangzhou, China) were used in the migration assays. After being culture for 24 h at 37°C, the cells (5 × 10^4^) were suspended in serum-free medium and added to the upper chamber and 10% FBS-medium was added in the lower chamber. After incubation for 24 h, the cells without migration in the upper chamber were carefully removed, and the migratory cells on the lower surface of the membrane were fixed and stained with crystal violet (*C_25_H_30_ClN_3_*). The number of migration cells was counted under a light microscope.

### Animal Models

A549 cells, A549 cells transfected with pcDNA3.1-Trop2, H1299 cells, and H1299 cells transfected with pcDNA3.1-Trop2 (2 × 10^6^ cells/mouse) were implanted subcutaneously in BALB/c nude mice. When the tumor volumes reached ~50 mm^3^, the mice were divided into three groups (Ctrl group, TCO group, and TCO+Trop2 group). The mice were intraperitoneally injected with TCO (50 mg/kg/day) or 0.1 ml of vehicle (10% ricinus oil, 5% DMSO, 10% ethanol, 75% physiological saline), respectively. The tumor volumes were monitored every 3 days using a handheld imaging device (TM900; Peira, Belgium). All animal study was approved by ethics committee of Hainan Medical University and abided by animal protocols (grant number: HY-2018-1004).

### Statistical Analysis

Statistical analysis was performed with GraphPad Prism 6.0 (GraphPad Software). Statistical differences between the two groups were performed by the Student’s t-test. Data were shown as means ± s.d. and statistical significance was designated as follows: *P < 0.05, **P < 0.01, and ***P < 0.001.

## Results

### Toxicarioside O Inhibits Cell Proliferation and Induces Apoptosis in Lung Cancer Cells

**Figure 1:**
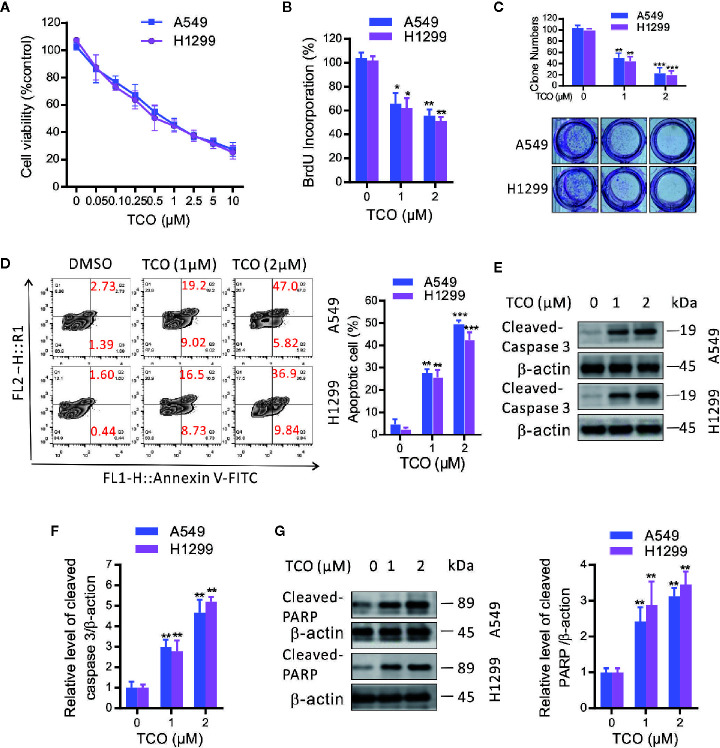
Toxicarioside O inhibits cell proliferation and induces apoptosis in lung cancer cells. **(A)** Cell viability was determined by MTT assay in A549 and H1299 cells. **(B)** Cell proliferation was determined by BrdU labeling in A549 and H1299 cells. **(C)** Cell proliferation was analyzed by colony formation assay in A549 and H1299 cells. **(D)** The apoptosis rate was analyzed by flow cytometry in A549 and H1299 cells. **(E, F)** Immunoblot analysis of caspase-3 in A549 and H1299 cells. **(G)** Immunoblot analysis of PARP in A549 and H1299 cells. The experiment was repeated three times. *P < 0.05, **P < 0.01, ***P < 0.001.

To investigate the effect of TCO on lung cancer cells, we examined the cell viability by MTT assay. As shown in **Figure 1A**, TCO treatment decreased the cell viability in a dose-dependent manner in both cell lines. Consistently, BrdU assay showed that cell growth was reduced under TCO treatment (**Figure 1B**). Moreover, TCO markedly inhibited cell proliferation in lung cancer cells, as evidenced by reduced colony formations (**Figure 1C**). Taken together, our findings indicated that TCO inhibits cell proliferation in lung cancer cells.

To determine whether TCO induces apoptosis in lung cancer cells, we examined apoptosis by flow cytometry following Annexin V-FITC and propidiumiodide (PI) staining. As shown in **Figure 1D**, TCO treatment for 24 h significantly increased the ratio of apoptotic cells in both A549 and H1299 cells. Consistent with this observation, we observed increased the levels of cleaved caspase-3 and cleaved PARP in TCO-treated cells (**Figures 1E–G**). Taken together, our findings demonstrated that TCO induces apoptosis in lung cancer cells.

### Toxicarioside O Suppresses EMT and Inhibits Cell Migration in Lung Cancer Cells

**Figure 2:**
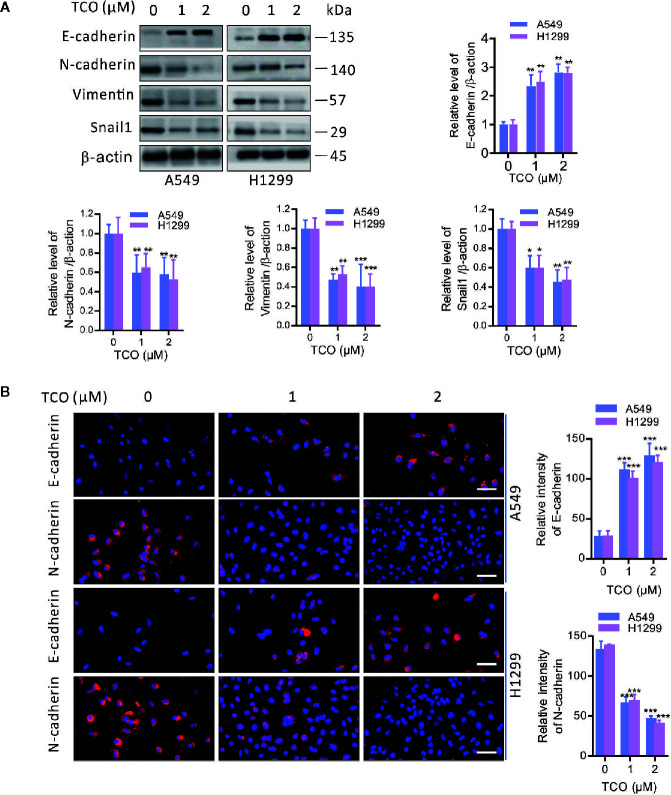
Toxicarioside O suppresses EMT in lung cancer cells. **(A)** Immunoblot analysis of E-cadherin, N-cadherin, Vimentin, and Snail1in A549 and H1299 cells. **(B)** Immunofluorescent staining for E-cadherin and N-cadherin in A549 and H1299 cells. Relative fluorescence intensity was analyzed by ImageJ. Scale bars, 50 mm. The experiment was repeated three times. *P < 0.05, **P < 0.01, ***P < 0.001.

To determine whether epithelial-mesenchymal transition (EMT) is involved in TCO-mediated antitumor effect in lung cancer cells, we examined the expressions of E-cadherin, N-cadherin, Vimentin, and Snail1 in TCO-treated cells. As shown in **Figure 2A**, TCO treatment significantly increased E-cadherin and decreased N-cadherin, Vimentin, and Snail1 expression in both cells by immunoblot analysis. Consistently, immunofluorescence staining assays showed that TCO increased E-cadherin and decreased N-cadherin expression in both cells (**Figure 2B**). Taken together, these results indicated that TCO suppresses EMT in lung cancer cells.

**Figure 3:**
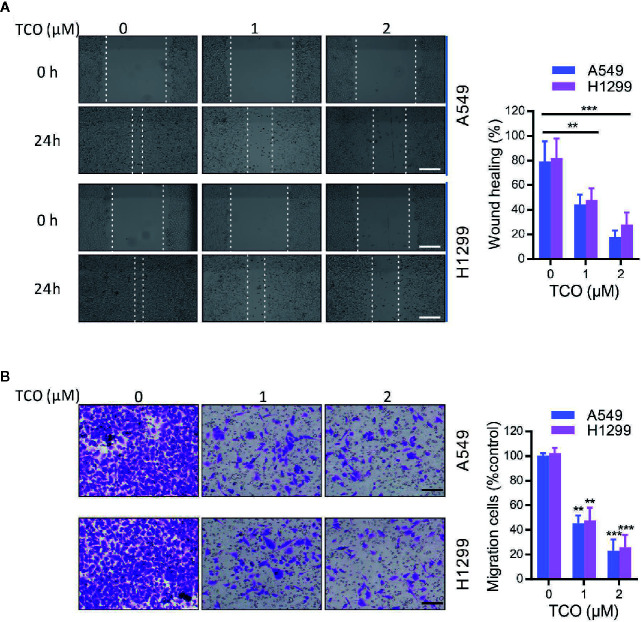
Toxicarioside O inhibits cell migration in lung cancer cells. **(A)** Representative images of wound healing assay of A549 and H1299 cells. Scale bars, 200 mm. **(B)** Migration assays in A549 and H1299 cells. Scale bars, 200 mm. The experiment was repeated three times. **P < 0.01, ***P < 0.001.

To validate whether TCO inhibits cell migration in lung cancer cells, we examined the cell migration viability by wound healing assay. As shown in **Figure 3A**, TCO treatment significantly suppressed cell migration in both cells. Consistently, transwell migration assay showed that TCO treatment significantly inhibited cell migration in lung cancer cells (**Figure 3B**). Collectively, these results indicated that TCO inhibits cell migration in lung cancer cells.

### Toxicarioside O Inhibits the PI3K/Akt Pathway in Lung Cancer Cells

**Figure 4:**
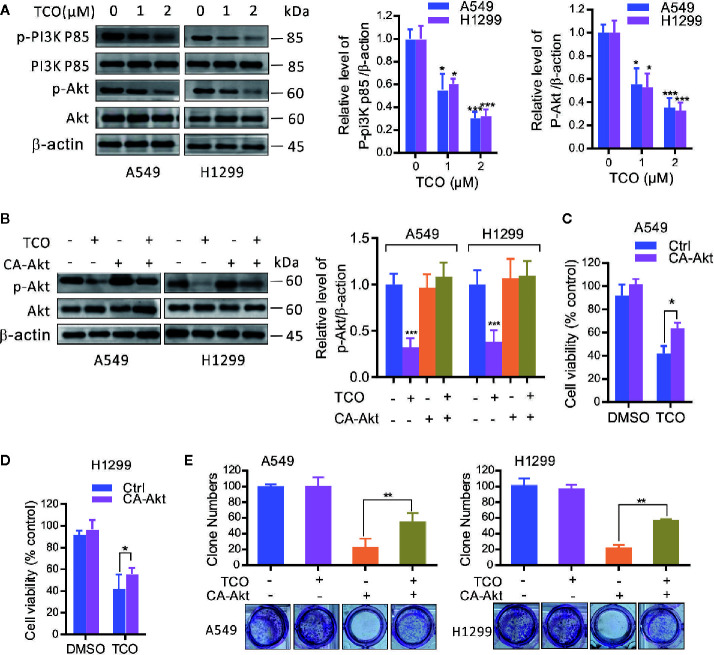
Toxicarioside O inhibits the PI3K/Aktpathway in lung cancer cells. **(A)** Immunoblot analysis of phosphorylation of Akt (Ser473) and PI3Kp85 (Y607) in A549 and H1299 cells. Total Akt and PI3K p85 expression served as an internal control. **(B)** Cells were transfected with an empty vector or constitutively active CA-Akt for 48 h, and then cells were treated with 2 μM of TCO for another 24 h. The p-Akt was determined by immunoblotting. **(C, D)** Cell viability was determined by MTT assay in A549 and H1299 cells stably expressing CA-Akt. **(E)** Cell proliferation was determined by colony formation assay in A549 and H1299 cells stably expressing CA-Akt. The experiment was repeated three times. *P < 0.05, **P < 0.01, ***P < 0.001.

To determine whether the PI3K/Akt pathway is involved in TCO-mediated antitumor effect in lung cancer cells, we examined the phosphorylation levels of Akt (Ser473) and PI3Kp85 (Y607). As shown in **Figure 4A**, TCO markedly decreased the phosphorylation levels of Akt and PI3Kp85, suggesting that TCO inhibits the PI3K/Akt pathway in lung cancer cells. Moreover, we expressed a constitutively active form of Akt (CA-Akt) to restore TCO-induced inhibition of the PI3K/Akt pathway (**Figure 4B**). As shown in **Figures 4C, D**, Akt activation significantly restored cell viability in TCO-treated cells. Consistently, similar results were observed by colony formation assay (**Figure 4E**). Taken together, these results indicated that TCO suppresses cell proliferation in lung cancer cells by inhibition of the PI3K/Akt pathway.

### Toxicarioside O Decreases Trop2 Expression in Lung Cancer Cells

**Figure 5:**
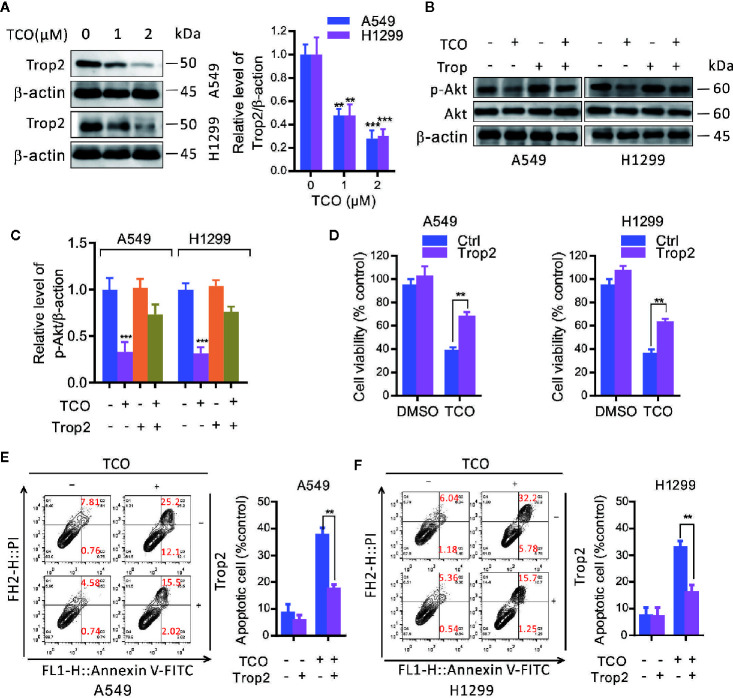
Toxicarioside O inhibits cell proliferation and promotes apoptosis by downregulation of Trop2 in lung cancer cells. **(A)** Immunoblot analysis of Trop2 in A549 and H1299 cells. **(B, C)** Cells were transfected with an empty vector or transfected with pcDNA3.1-Trop2 for 48 h, and then cells were treated with 2 μM of TCO for another 24 h. The p-Akt was determined by immunoblotting. **(D)** Cell viability was determined by MTT assays in A549 and H1299 cells stably expressing Trop2. **(E, F)** The apoptosis rate was analyzed by flow cytometry in A549 and H1299 cells stably expressing Trop2. The experiment was repeated three times. **P < 0.01, ***P < 0.001.

To determine whether trophoblast cell surface antigen 2 (Trop2) is involved in TCO-mediated antitumor effect in lung cancer cells, we firstly examined the expression of Trop2 in TCO-treated cells. As shown in **Figure 5A**, TCO treatment markedly decreased the expression of Trop2 in lung cancer cells. Overexpression of Trop2 reverted the phosphorylation levels of Akt (Ser473) in TCO-treated cells (**Figures 5B, C** **)**. We observed that Trop2 overexpression restored cell viability in TCO-treated cells by MTT assay (**Figure 5D**). Moreover, Trop2 overexpression obviously rescued TCO-induced apoptosis (**Figures 5E, F** **)**. Collectively, these results demonstrated that TCO inhibits cell proliferation and promotes apoptosis in lung cancer cells by downregulation of Trop2.

**Figure 6:**
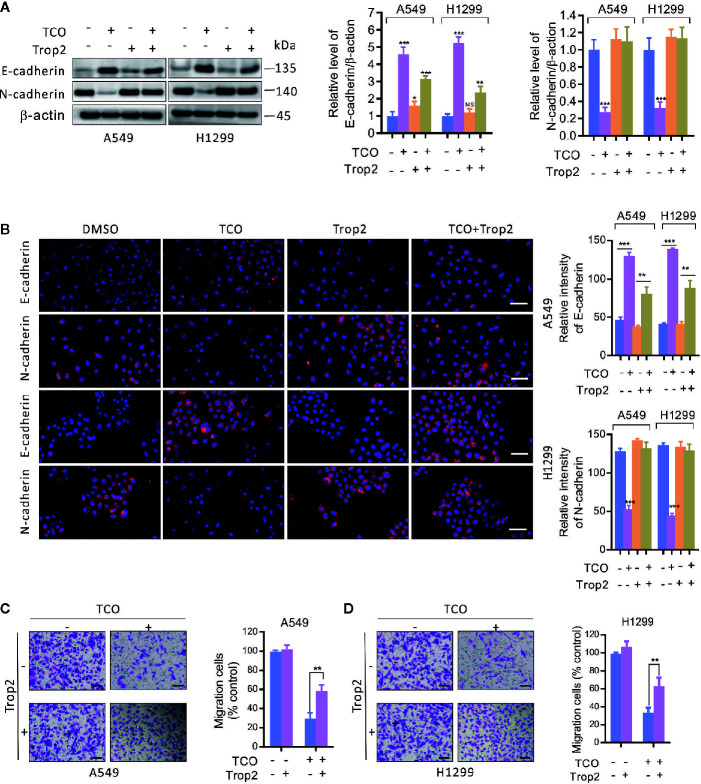
Toxicarioside O suppresses EMT and inhibits cell migration by downregulation of Trop2 in lung cancer cells. **(A)** Cells were transfected with an empty vector or transfected with pcDNA3.1-Trop2 for 48 h, and then cells were treated with 2 μM of TCO for another 24 h. E-cadherin and N-cadherin were determined by immunoblotting. **(B)** Immunofluorescent staining for E-cadherin and N-cadherin in A549 and H1299 cells stably expressing Trop2. Relative fluorescence intensity was analyzed by ImageJ. Scale bars, 50 mm. **(C, D)** Migration assay in A549 and H1299 cells stably expressing Trop2. Scale bars, 200 mm. The experiment was repeated three times. **P < 0.01, ***P < 0.001. NS, No Significant.

To determine whether Trop2 is involved in TCO-mediated EMT suppression in lung cancer cells, we overexpressed Trop2 and examined the expressions of E-cadherin and N-cadherin. As shown in **Figure 6A**, overexpression of Trop2 markedly inhibits E-cadherin expression but promotes N-cadherin expression in TCO-treated cells. Consistently, similar results were observed by immunofluorescence staining assay (**Figure 6B**). Moreover, we found that Trop2 overexpression markedly rescued TCO-mediated migration inhibition (**Figures 6C, D** **)**. Taken together, these results indicated that TCO suppressed EMT in lung cancer cells by downregulation of Trop2.

### Toxicarioside O Inhibits Tumor Growth *In Vivo*

**Figure 7:**
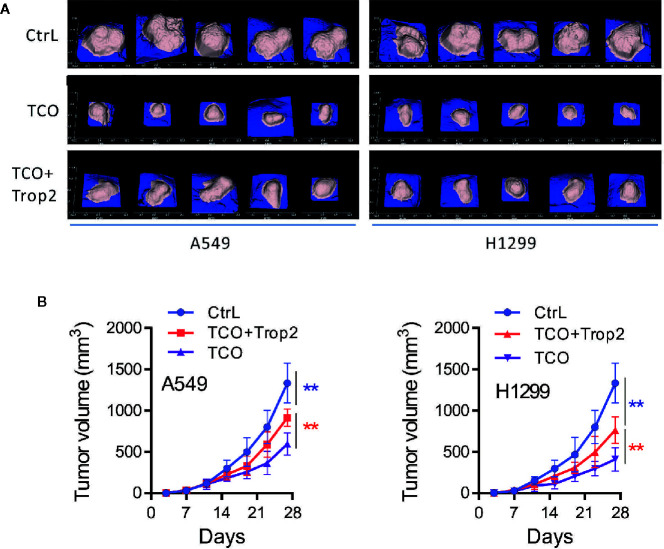
Toxicarioside O inhibits tumor growth *in vivo*. A549 cells, A549 cells transfected with pcDNA3.1-Trop2, H1299 cells and H1299 cells transfected with pcDNA3.1-Trop2 (2 × 10^6^ cells/mouse) were implanted subcutaneously in BALB/c nude mice. When the tumor volume was palpable (~50 mm^3^ at day 6), mice were treated with indicated formulations every 2 days for five total treatments. **(A)** Images of tumor masses at day 28 in each mouse. **(B)** Tumor volumes at different time points. The experiment was repeated three times. **P < 0.01.

To evaluate the effect of TCO on lung cancer growth *in vivo*, A549 andcA549 cells transfected with pcDNA3.1-Trop2 (or H1299 and H1299 cells transfected with pcDNA3.1-Trop2) were implanted subcutaneously in BALB/c nude mice and treated with TCO or DMSO. The images of tumor masses (five in each group) were observed as shown in **Figure 7A**. Compared to the Ctrl group, the tumors in TCO and TCO+Trop2 groups grew significantly slower, and the TCO group grew the slowest, respectively (**Figure 7B**). These data indicated that TCO significantly inhibited the growth of lung cancer *in vivo*, and Trop2 overexpression markedly rescued TCO-mediated tumor growth inhibition.

## Discussion

In recent years, cardenolides, which is traditionally used in the management of congestive heart failure and arrhythmia, have got much attention due to their antitumor effect (2, 3). Toxicarioside O (TCO) is one of the cardenolides isolated from the seeds of *Antiaris toxicaria* that exhibits potential anticancer activities. TCO significantly inhibits cell proliferation in SMMC-7721 and K562 (1). We have previously demonstrated that TCO promotes apoptotic cell death and induces protective autophagy in colorectal cancer cells (6). In this study, we demonstrated that TCO markedly inhibits cell proliferation, migration, and epithelial-to-mesenchymal transition (EMT) in lung cancer cells. Moreover, we revealed that TCO inhibits cell proliferation and migration by downregulation of trophoblast cell surface antigen 2 (Trop2), indicating that Trop2 plays a critical role in TCO-mediated antitumor effect in lung cancer cells.

Trop2, also known as human tumor-associated calcium signal transducer 2 (Tacstd2), is a surface glycoprotein upregulated in various cancer cells (7, 8). Trop2 overexpression is associated with poor survival in human solid tumors, and it has been considered as a potential target for anticancer therapy (12, 14). Blockade of Trop2 by anti-Trop2 antibodies exhibits potential anticancer activities (24, 25), and suppression of Trop2 by a natural product of Curcumin significantly inhibits cell proliferation and motility in bladder cancer cells (26). Additionally, RNAi-mediated Trop2 loss markedly triggers antitumor response in some cancer cell lines (27, 28). Consistent with these findings, here we demonstrated that downregulation of Trop2 by a natural product of TCO significantly inhibits cell proliferation and EMT in lung cancer cells. Trop2 is a transmembrane glycoprotein encoded by the Tacstd2 gene and its overexpression is found in several types of cancer. Although several transcription factors have been identified to regulate its expression, the mechanisms of Trop2 regulation are still unclear (14, 29, 30). In this study, we demonstrated that TCO downregulates Trop2 expression in protein level in lung cancer cells, but how to modulate Trop2 expression by TCO needs further study.

Numerous intracellular signaling pathways mediated by Trop2 are essential for the growth of human cancer cells including PI3K/Akt pathway. Trop2 overexpression activates the PI3K/Akt pathway, and promotes cell proliferation and invasion (31, 32). In contrast, blockage of Trop2 by antibodies or downregulation of Trop2 by natural products suppresses cell proliferation in cancer cells through inhibition of PI3K/Akt pathway (12, 33). In this study, our results demonstrated that TCO suppresses cell proliferation in lung cancer cells by inhibition of the PI3K/Akt pathway. Furthermore, we revealed that TCO inhibits the PI3K/Akt pathway by downregulation of Trop2.

EMT is referred to changes in cell phenotypes from epithelial to mesenchymal states which mediates cancer cell mobility, invasion, and drug resistance (17–19). Accumulating evidence suggested that Trop2 induces EMT and promotes cancer progression in several types of cancer. Trop2 has been regarded as a potential biomarker for the promotion of EMT in human breast cancer (22). Trop2 overexpression promotes migration and metastasis of gallbladder cancer cells by inducing EMT and knockdown of Trop2 suppresses EMT and inhibits migration in endometrial cancer (31, 33). In this study, our results indicated that TCO suppresses EMT and inhibits cell migration by downregulation of Trop2 in lung cancer cells, but the molecular mechanism of EMT regulation will be further explored in the future.

In summary, we demonstrated that TCO suppresses cell proliferation and EMT in lung cancer cells. We further revealed that TCO decreases Trop2 expression, leading to inhibition of the PI3K/Akt pathway and EMT suppression. Overexpression of Trop2 rescues TCO-mediated inhibition of cell proliferation and EMT in lung cancer cells. Our findings provide novel insights into the molecular basis for the anti-cancer effect of TCO, and demonstrate that TCO is a potential anticancer agent against lung cancer.

## Data Availability Statement

The original contributions presented in the study are included in the article/supplementary materials. Further inquiries can be directed to the corresponding authors.

## Ethics Statement

The animal study was reviewed and approved by the ethics committee of Hainan Medical University (grant number: HY-2018-1004).

## Author Contributions

W-PZ, F-YH, and S-ZD designed, carried out the experiments, analyzed the data, and prepared the draft of manuscript. J-YW, Y-YL, and YS conducted the experiments and provided materials for biological assays. G-HT and Y-HH conceived the idea, supervised all research, and revised the manuscript. All authors contributed to the article and approved the submitted version.

## Funding

This work was funded by the Natural Science Foundation of Hainan Province (819MS062) and the National Natural Science Foundation of China (81860429, 81660004, 81760634, 81860650, and 81673346).

## Conflict of Interest

The authors declare that the research was conducted in the absence of any commercial or financial relationships that could be construed as a potential conflict of interest.
